# Overlooked and undertreated: gendered ageism in primary care management of eating disorders

**DOI:** 10.1186/s12939-025-02702-0

**Published:** 2025-12-30

**Authors:** Theresa Kohestani, Pamela Otto, Hanna Köttl

**Affiliations:** 1 Fonds Soziales Wien, Vienna, Austria; 2 Department of Health Sciences, IMC University of Applied Sciences Krems, Krems, Austria

**Keywords:** Eating disorders, Older adults, General practitioner, Diagnosis, Treatment, Gendered ageism

## Abstract

**Background:**

Eating disorders (EDs) in older adults remain underrecognised due to persistent stereotypes framing them as conditions of youth. This study investigates how general practitioners (GPs) in Austria perceive, diagnose, and manage EDs in patients aged 65 and over, and explores the potential role of ageism in the context of clinical decision-making.

**Methods:**

A vignette-based, semi-structured interview design was used with nine Austrian GPs experienced in treating older patients. The vignette described an older woman presenting with symptoms of Anorexia Nervosa. Data were analysed using a content-structuring qualitative approach, identifying patterns in awareness, diagnostic reasoning, and treatment practices.

**Results:**

Two overarching themes emerged: (1) Awareness and knowledge of EDs in later life and (2) Diagnostics, treatment, and differential diagnosis. Most GPs reported little familiarity with EDs in older adults, often attributing appetite or weight loss to ageing or somatic illness. Gendered stereotypes shaped perceptions, with older women viewed as less concerned with appearance and older men’s EDs largely invisible. Diagnostic challenges included the absence of validated screening tools for older populations, symptom masking by multimorbidity, and reliance on physical rather than psychiatric explanations. Treatment pathways varied, ranging from interdisciplinary collaboration to psychiatric referral or antidepressant initiation, often targeting depression rather than the ED itself.

**Conclusions:**

The underrecognition of EDs in older adults reflects structural gaps, ageist and gendered assumptions, and lack of tailored clinical guidelines. Age- and gender-sensitive screening tools, specific treatment pathways, targeted GP training, and public health campaigns are needed to improve detection and care for this underserved population.

**Supplementary Information:**

The online version contains supplementary material available at 10.1186/s12939-025-02702-0.

## Background

Eating disorders (EDs) have long been stereotyped as illnesses that primarily affect young, white, affluent women [1–3]. This narrow and outdated perception has contributed to the significant underrecognition and underdiagnosis of EDs among other demographic groups, including individuals of different ages, genders, ethnicities, and socioeconomic backgrounds [1, 3–5].

According to the *International Classification of Diseases, 11th Revision* [6], EDs are classified as a group of mental and behavioural conditions marked by significant disturbances in eating behaviour, appetite regulation, and food intake, frequently accompanied by both physiological and psychological impairments. In some cases, these disturbances can have life-threatening consequences, particularly when energy intake is restricted below the body’s requirements [7]. EDs include diagnoses such as Anorexia Nervosa, Bulimia Nervosa, and Binge Eating Disorder, among others, as per the Diagnostic and Statistical Manual of Mental Disorders 5 (DSM 5) [8]. Although research has traditionally concentrated on adolescents and young adults, an increasing body of evidence underscores the persistence and clinical significance of these conditions across the adult life span, including in later life (e.g., [9–12]).

Symptoms such as weight loss, appetite changes, or disrupted eating patterns are often attributed to normative ageing processes rather than recognised as potential indicators of underlying mental health conditions [13, 14]. This tendency reflects broader manifestations of ageism in healthcare, understood as the stereotyping, prejudice, and discrimination directed toward individuals based on their age [15]. Older adults, despite forming an increasingly large segment of healthcare users, often receive less diagnostic scrutiny, less intensive treatment, and more limited access to preventive care [16–18]. Furthermore, clinical guidelines tailored to their specific physical and psychological needs remain underdeveloped [19, 20]. Evidence suggests that such systemic biases extend to the treatment of EDs: older women with EDs report substantial gaps in service provision, often encountering ageist attitudes, a lack of age-appropriate resources, and dismissive responses from healthcare professionals [14]. Such systemic age-related biases likely play a role in the continued underrecognition and inadequate treatment of EDs among older adults - an issue that has received limited scholarly attention to date.

### The prevalence of EDs: a lifespan approach

Contrary to long-held stereotypes, EDs are not confined to adolescence or young adulthood [21–23]. Mounting evidence underscores that these conditions can persist across the lifespan and may even re-emerge in later life, particularly during periods of major transition such as menopause, bereavement, or retirement [9, 10, 12]. These findings challenge the misconception that ageing offers immunity against body image concerns or disordered eating behaviours [23].

EDs are most commonly diagnosed during adolescence and young adulthood, with peak onset between ages 16 and 25 [24, 25]. Onset after age 40 is considered late-onset and remains under-recognized in both clinical and research contexts [10, 26]. Longitudinal research has shown that symptoms often begin in adolescence and may persist for decades. For instance, a 21-year follow-up study in Germany found that only 51% of individuals fully recovered, with a significant proportion experiencing relapse much later in life [27].

Among women aged 40 and older, prevalence estimates for full-syndrome EDs range from 2.1% to 7.7% [12, 28]. In a Canadian cohort, 2.6% of women aged 50–64 and 1.8% of those aged 65 and older met diagnostic criteria for an ED [29]. Similarly, Lapid et al. [26] reported a prevalence of 3.8% among community-dwelling women aged 60–70. Although less frequently studied, men are also affected by EDs across the lifespan. Prevalence estimates among older men range from less than 1% to approximately 3% [12, 28]. For men in particular, ED symptoms, such as weight loss or changes in eating behaviour, are often misattributed to normative ageing processes, contributing to underdiagnosis and delayed intervention [13, 14, 30].

While comprehensive prevalence data for individuals aged 65 and over remain limited, there are some indications for concern: In a US population-based survey of over 4,000 women aged 25 to 45, nearly one-third reported engaging in purging behaviours to control their weight, and over 40% had used diet pills [31]. Among Austrian women aged 60 to 70 in a nonclinical sample, more than 80% reported using weight control methods, and 60% expressed dissatisfaction with their bodies [11]. Importantly, these concerns were reported not only by overweight individuals but also by women of normal or underweight status.

### Etiology, risk factors and comorbidities

The etiology of EDs in older adults reflects a complex interplay of psychological, biological, and sociocultural factors [32]. While many risk factors mirror those found in younger populations, such as perfectionism, low self-esteem, and internalized thin ideals [33, 34], older adults also face age-specific stressors that may trigger or exacerbate disordered eating.

Psychosocial transitions like retirement, bereavement, caregiving responsibilities, and declining health can act as catalysts for ED onset or relapse [10, 12]. Biological changes associated with ageing - such as hormonal shifts during menopause and testosterone decline - have also been identified as contributing factors [10, 30].

Moreover, depression and anxiety are commonly comorbid with late-life EDs [35], while obsessive-compulsive personality disorders are associated with both early and late onset [36]. These psychiatric conditions may complicate diagnosis and treatment, particularly in older adults where symptoms may overlap with other age-related (mental) health concerns.

Body dissatisfaction remains a stable risk factor across the lifespan [9, 23] and is not limited to individuals with overweight or obesity. These concerns are often compounded by societal ageism and the invisibility of older bodies in mainstream representations of health and beauty [37].

### Treatment approaches

Research on the treatment of EDs in older adults remains limited and methodologically weak. A systematic review by Mulchandani et al. (2021) examined 35 studies involving individuals aged 66 to 94 years, of whom 84.6% were female. The most prevalent diagnosis among older adults receiving treatment for EDs was Anorexia Nervosa, accounting for 84.6% of the cases examined. Most participants received treatment, with hospital-based care being common. In cases reporting improvement, a multidimensional approach - combining hospitalization, psychotherapy, and pharmacological therapy - was most effective. Overall, 79.5% of treated individuals showed improvement, while 20.5% relapsed or died due to complications.

Complementing these findings, Lapid et al. (2010) analyzed 48 published cases of EDs in individuals aged 50 and older. The majority were women (88%), and Anorexia Nervosa was the most frequent diagnosis (81%). Treatment was provided in 75% of cases, with the most successful outcomes reported in those receiving combined behavioral and pharmacologic interventions. Electroconvulsive therapy (ECT), though rare, was also effective in the few cases where it was used. Despite these interventions, only 42% of cases showed sustained improvement, and 21% resulted in death due to ED-related complications. These results underscore the need for early recognition and tailored, multidisciplinary treatment strategies in older populations.

Although evidence supports the efficacy of psychological interventions for older adults, studies consistently show that they are less likely to receive psychotherapeutic treatment than younger individuals and are instead more often treated pharmacologically [38, 39] - a pattern that may also extend to the treatment and care of older persons with EDs.

### General practitioners’ role in detecting and treating EDs in older persons

Despite the high morbidity and mortality associated with EDs in older adults, these conditions often remain undetected and untreated in this population [23, 26, 28, 40]. General practitioners (GPs) are frequently the first healthcare professionals to encounter older adults with EDs, placing them in a crucial position for early identification and intervention.

However, research indicates limited awareness of EDs among healthcare professionals, including GPs [41]. Symptoms are often misinterpreted or attributed primarily to normal age-related changes, further delaying diagnosis [42]. While several qualitative and quantitative studies have explored healthcare professionals’ awareness, experiences, and perceptions regarding the diagnosis and treatment of EDs (e.g., [43–45]), to date, no research has explicitly taken a lifespan approach to examine how GPs recognise and manage EDs in older adults, including how ageist assumptions may influence clinical judgments.

Given the growing recognition of EDs as a significant concern among older populations [12], it is essential to strengthen GPs’ awareness and competencies in identifying and treating these conditions until old age. Targeted professional education, enhanced screening practices, and the development of age-sensitive clinical guidelines are critical steps toward improving care outcomes for this often-overlooked patient group. The primary aim of this study is hence to explore how GPs in Austria perceive and assess older patients (aged 65 and above) who present with symptoms indicative of an ED, and to identify potential treatment pathways. Specifically, the study seeks to: (a) examine GPs’ awareness and knowledge of EDs in later life, and (b) investigate diagnostic practices and treatment paths, including the consideration and exclusion of differential diagnoses in clinical decision-making.

## Methods

A vignette-based approach was employed and integrated into semi-structured expert interviews, allowing participants to reflect on and discuss a standardized case in-depth. Phenomenology was chosen as it allows for rich descriptions of lived experience, clinical judgment, and meaning-making processes, particularly useful in understudied areas like late-life EDs [46].

Participants were recruited using purposive sampling, targeting GPs in Austria who currently or previously treated patients aged 65 or older. Inclusion criteria were (a) Working or having worked as a GP in Austria and (b) Clinical experience with individuals aged 65 and over. Initial attempts to recruit through newsletters from Austria’s regional medical chambers were declined due to limited resources. As an alternative, recruitment was carried out via the public physician directories on the medical chamber websites for Vienna and Lower Austria. Using web scraping techniques [47], contact details of GPs with listed email addresses were extracted and compiled into an excel database. Recruitment emails were distributed using Microsoft Word’s mail merge function. A total of 166 emails were sent in Vienna and 869 in Lower Austria between late May and early June 2024. Twelve GPs expressed interest; three could not be reached during scheduling, resulting in nine participating in the interviews. One participant was recruited via snowball sampling, while the others responded directly to the recruitment emails.

All participants were provided with a case vignette depicting a hypothetical older woman exhibiting symptoms consistent with Anorexia Nervosa (Attachment 1). The vignette was based on established case studies [42, 48] and aligned with ICD-11 diagnostic criteria. The vignette was refined with feedback from experts in ageing and EDs to ensure clinical accuracy and contextual relevance. The vignette method was selected for its ability to standardize participant input while allowing for in-depth discussion of diagnostic reasoning and treatment preferences [49]. Previous research has shown that vignettes can effectively expose age-based stereotypes and clinical decision-making biases in healthcare [50, 51]. Participants were invited to reflect on the vignette and discuss possible etiological assumptions, diagnostic considerations, assessment strategies, and treatment pathways. Although the vignette itself did not specify a diagnosis, the recruitment materials explicitly indicated that the study focused on EDs.

Interview data were collected through semi-structured expert interviews (by T.K.), which allow for the systematic yet flexible exploration of expert knowledge and reasoning [52, 53]. Interviews lasted between 45 and 70 minutes and were conducted according to participant preference - either in person at their workplace, by telephone, or via Microsoft Teams. Interviews were conducted one-on-one, with only the participant and the interviewer present. Following each interview, detailed field notes were recorded to capture contextual observations and initial reflections. Each interview was audio-recorded with consent and later transcribed verbatim using Microsoft Word’s transcription function. All interview transcripts were manually reviewed for accuracy to ensure consistency with the corresponding audio recordings.

The analysis followed the content-structuring qualitative approach described by Kuckartz and Rädiker [54], which combines deductive and inductive analytical strategies. Transcripts were imported into MAXQDA 24 for systematic coding and data management. The coding process focused on identifying patterns in how GPs perceived and described EDs in older adult patients, as well as how they approached diagnostic procedures, including the exclusion of differential diagnoses. Particular emphasis was placed on examining whether and how age-related assumptions influenced both the recognition and clinical assessment of ED symptoms. Guided by predefined analytical dimensions derived from prior research and the study objectives, the data were first analyzed deductively. This process led to the identification of two main categories. As no additional main categories emerged during the analysis, inductive coding was subsequently applied to identify finer nuances and explore emergent subthemes. This combination of deductive and inductive approaches allowed for both structured analysis and openness to unexpected insights. Evolving categories and subcategories were collaboratively refined through regular team meetings, fostering collective reflection and enhancing analytical depth. While the first author (T.K.) led the initial transcript analysis, the second and third researchers contributed through ongoing discussions, offering critical feedback and interpretive input. These collaborative practices supported the development of a coherent coding framework and enhanced the trustworthiness [55] of the findings.

Theoretical sensitivity [56] was strengthened by the authors’ diverse academic backgrounds. All authors identify as female and bring diverse professional and academic expertise to the study: T.K. in nutrition and dietetics with a focus on geriatrics, P.O. in nursing science with extensive experience in EDs, and H.K. in occupational therapy and gerontology. The researchers shared a prior interest in clinical decision-making, ageing, and mental health, shaped by their respective disciplines.While no pre-existing relationships with participants existed, the researchers acknowledged that their disciplinary backgrounds and personal engagement with the topic may have influenced both data collection and interpretation. Participants were informed that the study was conducted as part of a master’s thesis, that the interviewer was a qualified dietitian, and that the research focused on EDs in older age. Reflexive practices were applied throughout the study to mitigate potential bias and enhance transparency. During the pilot phase, the interview guide was tested on two people. One interviewee had a background in allied health professions, while the other was in their penultimate semester of medical studies and has since graduated. The Consolidated Criteria for Reporting Qualitative Research (COREQ) checklist [57] was applied to guide the reporting of study design, data collection, analysis, and researcher reflexivity (Attachment 2).

The study was reviewed by the Lower Austrian Ethics Committee, which granted a waiver of ethical approval (GS3-EK-12/804–2024). All participants provided written informed consent after being briefed on the study’s aims, data protection measures, and their right to withdraw at any time. Anonymity and confidentiality were strictly maintained, and all data were stored securely on encrypted devices.

## Results

Table 1 presents a description of the characteristics of the nine GPs who met the inclusion criteria for the study. To safeguard the subjects’ anonymity, their names were replaced by pseudonyms. The participants’ ages ranged from 36 to 72 years, with an average age of 50 years. Four identified as female and five as male. Apart from one participant, all had undergone supplementary training or specialised medical training in addition to their general practice training. The participants of the study were employed in the following professional settings: five in private practices, two in group practices, and two doctors each in private practices located in medical or health centres.

**Table 1:** Expert characteristics

Experts	Age	Gender	GP since	Work setting	Additional Training
A1	38	male	2018	Individual practice in a medical centre	MSc in preventive medicine
A2	39	female	2024	Group practice	Palliative care, special pain therapy, occupational medicine, emergency doctor
A3	42	female	2022	Group practice	Anaesthesia, emergency physician
A4	62	female	1995	Individual practice	-
A5	38	male	2023	Individual practice	Neurosurgery
A6	72	male	1982	Individual practice	Psychotherapy, auditor and trainer for quality risk management in healthcare
A7	65	female	2003	Individual practice	Internal medicine
A8	65	male	1990	Individual practice	Psychotherapy, supervision, emergency physician
A9	36	male	2020	Individual practice in a health centre	Neurology

Note. GP = General practitioner

As highlighted in Table 2, the qualitative analysis of GPs’ perspectives, perceptions, and clinical attitudes toward the diagnosis and treatment of EDs in individuals aged 65 and older identified two deductively formed main categories: (1) Awareness of EDs in older age and (2) Diagnostics, treatment, and differential diagnosis. The first category, comprising three subcategories, reflects participants’ awareness, clinical experience, stereotypical assumptions, and risk assessments related to EDs across age groups. The second category, with four subcategories, addresses diagnostics, treatment paths, and differential diagnosis, including diagnostic tools, approaches for older adults, age-related differences, and the challenges and limitations of diagnosis and treatment in later life.

**Table 2:** Systematics of categories

Main category	Subcategory
Awareness and knowledge of EDs in older age (d)	Professional experience and awareness (i)
	Preoccupations and stereotypical images of ageing (i)
	Risk assessment across ages (i)
Diagnostics, treatment paths, and differential diagnosis (d)	Tools to support diagnostics (i)
	Treatment approaches in older adults (i)
	Differences in diagnostics and treatment based on age (i)
	Challenges and limitations in diagnosis and treatment of older adults (i)

Note: The deductively developed main categories are marked with a “d”, and the inductively developed subcategories are marked with an “i”

## Awareness and knowledge of EDs in older age

This category explores GPs’ awareness, clinical impressions, and attitudes concerning EDs in older adults. Further, it considers how gendered assumptions and age-related stereotypes shape perceptions of prevalence, diagnostic salience, and treatment relevance. The findings indicate varying degrees of professional experience and conceptual clarity, with some participants expressing uncertainty or scepticism about the clinical significance of EDs in later life.

### Professional experience and awareness

Many participants reported limited familiarity with EDs in older populations and highlighted that these conditions were seldom considered in their routine clinical practice. While a few GPs demonstrated critical self-reflection regarding their own lack of awareness, others questioned the diagnostic validity of EDs in older patients altogether.

Because in older people you tend to think about it less, I find. What is clinically very clear, like anorexia, I always associate that with younger people, with women. Although there are men too, yes more and more. There’s definitely not enough awareness, so I kind of have to call myself out on that too. (A2)

I wouldn’t [think of anorexia nervosa] with the woman [refers to case vignette] - I know enough people like that when I think about my time in hospital. Retirement, a downturn in life in some way or a developmental crisis, loss of a partner, a lack of reorientation, helplessness - that this also has a somatizing effect on eating behaviour, I know that very well. Only I wouldn’t call it anorexia - and that’s where we have a problem with our specialists. There is a very cynical, but unfortunately true, saying from statisticians. They say, ‘You get what you measure!’ - meaning that if I look at it through the lens of eating disorders, then I will find it too. (A8).

Overall, most GPs indicated little or no prior experience diagnosing or managing EDs in older patients. Changes in appetite or weight were more commonly attributed to somatic illness, depression, or psychosocial transitions associated with ageing, rather than considered within a psychiatric framework such as anorexia nervosa or bulimia nervosa.

Oh God, I would say, almost at all, not at all, yes… Exactly, so that it was specifically like an eating disorder, i.e. loss of appetite in old age, but not now, in the sense of an eating disorder. Most of the time there was something physical behind it or a depression that got worse, but I don’t really have any experience with eating disorders. (A2)

Reflecting on the current clinical landscape, one expert described their perception of the prevalence and development of different EDs in later life, particularly noting the increasing visibility of some conditions over others:

So, anorexia in older age is still rare at the moment, but of course, it’s becoming more common. They’re getting older too - the young ones are growing old eventually - but there are already plenty of cases of binge eating disorder and also bulimia. But anorexia, from my perception, is still rather uncommon at this point. (A8)

Another GP with a specialization in diabetes estimated that she had treated 30 to 40 older women with EDs and suspected that many more cases remained undetected.

Experiences with male patients were limited, and while some acknowledged the possibility of overlooking EDs in older men, others maintained that they would detect such conditions regardless of gender:

A lot more women, I get a lot more women. No men. I have only had one anorexic man in my entire 30-year career and that was a young man. (A4)

Of course, I also saw men in the hospital who suffered from anorexia, but the majority are actually women. (A9)

So men, when it comes to anorexia and bulimia, not at all. The international situation is also very clear that this does not happen. But when it’s in the phase or - after 65 no more - but in the phase where it exists, I think this Adonis complex is called Biggerexia, where they prefer to sit in the fitness center for 10 h a day and build up their muscles and throw everything in. There are, there are quite a few. (A6)

### Preoccupations and stereotypical images of ageing

Several GPs expressed assumptions that may reflect age-biased perceptions, which appeared to influence how they interpreted symptoms and approached treatment decisions. These included views that older adults may be less motivated to pursue recovery, less concerned with their appearance, or inherently less treatable. One GP, for instance, described older patients as largely unwilling to engage in inpatient care, illustrating how such assumptions may shape therapeutic expectations and decisions:

The older ones, I think, you can forget about getting them out [into treatment]. I think that - at least the ones I know - you can forget it. Yes, for them, an antidepressant is already the upper limit (…). Yes, yes, exactly, they are already tired. They have already experienced a lot in life. They just don’t want to anymore; they don’t have the motivation either. (A3)

As another reason the same GP argues that older people have their routines and don’t like to change them:

Yes, and they’re just so caught up in their lives and have a lot of responsibilities. They have to look after the grandchildren or, I don’t know, water the garden every day - the kinds of jobs they have. They don’t really want to go away for three weeks for rehab, try something new and so on. That’s always exhausting for them. They just want to be left alone and not eat. (A3)

Some participants expressed the assumption that concerns related to body image and appearance - and the emotional suffering tied to them - are predominantly issues of younger people, while older adults are thought to neglect their appearance more than younger individuals:

I don’t know what experience you have, but I see the young a lot more, yes. They also have more suffering with their appearance than when they are older. (A4)

(…) The young people then have this body dysmorphic disorder or whatever. They always see themselves as too fat. Older people will probably neglect themselves more at some point. Yes, there is certainly a difference between them and well, the consequences are always the same… (A1)

There was also a tendency to normalize weight loss in older adults by attributing it to age-related physiological changes rather than pathology:

Yes, well, the young women are starving themselves to death. This is a psychiatric diagnosis, anorexia or bulimia, and older people often lose weight towards the end of their life with muscle loss and so on, so they tend to have less appetite. (A1)

### Risk assessment across ages

Perceived severity of EDs in older adults was often downplayed. Several GPs believed that being underweight in older age posed fewer health risks and could even be beneficial, leading to the assumption that treatment for EDs in this group is less necessary - contrasting sharply with the greater concern shown for EDs in younger individuals.

Because I think there are advantages to being thin in older age. The cancer rate drops significantly. The body, all the joints, everything is virtually relieved, so to speak. So yeah, I don’t think it’s as life-threatening as anorexia nervosa in a seventeen-year-old, of course. The [older] people have more osteoporosis and more broken bones, of course everyday life is more difficult to cope with, but I think it’s not that dramatic anymore. (A3)

The huge difference, of course, is that if someone is 65 plus with an eating disorder, then I no longer need to talk to them about food. They can survive, otherwise they would no longer be there. (A7)

These perspectives indicate a systematic underestimation of the clinical relevance of EDs in older adults, potentially resulting in underdiagnosis or lack of intervention.

## Diagnostics, treatment paths, and differential diagnosis

This category explores age-related differences in diagnosing and treating EDs, highlighting how patient age influences clinical recognition and management. It addresses the use of diagnostic tools, physician–patient communication, and the importance of multidisciplinary care, while also discussing challenges in distinguishing EDs from other conditions, especially in older adults.

### Tools to support diagnostics

Overall, a variety of perspectives and approaches emerged concerning the use of screening tools and questionnaires for EDs. Some physicians opt not to use instruments specifically designed for identifying these conditions. For instance, A4 relies instead on a general medical history questionnaire: *No, I don’t really need that. I also have a medical history questionnaire, just the standard one in my practice. (A4)*

Differences also emerged in physicians’ knowledge of and attitudes toward specific screening tools, with some expressing uncertainty about the available questionnaires and how to effectively apply them, indicating varying levels of familiarity and experience with these instruments.

I’m quite sure that there is something out there. Yes, I just don’t know right now what options exist. There are certainly some questionnaires where you can calculate some kind of score. Off the top of my head, I actually don’t have any documents or anything like that. (A5)

One physician described using the EAT-26 screening questionnaire in the past when it was a standard tool but expressed uncertainty about its current use. Concerns were also raised about the reliability of weight measurements in patients with EDs, while another physician reported relying mainly on BMI for diagnosis, acknowledging its limitations.

### Treatment approaches in older adults

Experts A2, A3, A4, A7, and A8 emphasized the importance of a holistic and interdisciplinary approach to treating EDs in older adults, which comprehensively addresses both physical and psychological aspects. Furthermore, GPs highlighted the significance of establishing a trusting therapeutic relationship and maintaining clear, transparent communication with affected individuals. The multidisciplinary approach, involving close collaboration with specialized professionals and dedicated treatment centers, is regarded as indispensable for effective care.

In cases where their own resources or competences were estimated as insufficient, experts A1, A4, and A5 recommended referring patients to specialists, particularly psychiatrists or psychotherapists.

Yes, well, for me it’s clear - I send them to the psychiatrist. I try to intervene somehow in gerontopsychiatry, because if it’s a genuine eating disorder, then with my complementary medicine I’m out of place anyway. And if these psychiatric issues are not being treated, then they probably won’t be able to gain weight anymore. (A1)

Opinions on the use of medication varied across physicians. Some supported the use of antidepressants: A*nd maybe start with Cipralex or something like that, just to begin. (A3).* Others were more cautious: *Medically, I am very reserved and careful with antidepressants, and they really have to be a classic endogenous* [depression without an identifiable cause] *case. (A6*).

There were differences regarding when and how the topic of ED was addressed directly. One expert was more cautious and preferred to build trust first:

Whether I brought up the topic then depends on how I feel and perceive the relationship with her. Whether I say something about it then or just strictly stick to what she presents - her complaints - and maybe address it later… maybe not even then, but in the next conversation, it depends on how it goes. (A7)

Another physician preferred to address the issue immediately:

… I would probably bring it up beforehand, that I think she has an eating-related issue. That we need to consider this in treatment, and well, depending on how she reacts, I would then refer her to psychotherapy or a psychiatrist, depending on how severe it is. (A3)

### Differences in diagnostics and treatment based on age

Some experts emphasize that EDs in older adults are often more difficult to identify, with A7 noting that symptoms are frequently obscured by other health issues or age-related changes.

(…) the symptomatology in older adults is much more obscured, overlapped by multimorbidity in old age. (…). Yes, I find it much harder to recognise. Exactly what we have already discussed, and especially with anorexia, the late-stage damages are in the foreground: osteoporosis, cardiac arrhythmias, kidney insufficiency, anaemia, and all the consequences these cause. (A7)

In contrast, some GPs perceive the core symptoms of EDs as largely similar across both younger and older patients. However, limited clinical experience with older adults can make it challenging to form definitive assessments in this group. Denial or concealment of symptoms is a common challenge regardless of age, with younger patients frequently insisting they are eating adequately - a behaviour that is also observed among older individuals.

Yes, and what unites them all, of course, is the denial. With the younger ones it’s: “Yes, Mom, that’s not true, I do eat enough.” And with the older ones it’s: “Oh, doctor, I eat three times a day.” And then you ask: “Well, how often do you cook for yourself during the week?” and then: “How often do you actually eat, and how much do you eat?” (A9)

A subset of experts reported that patient age does not influence the course of treatment.

Well, I think I would approach it in a similar way. My focus is always to first clarify the physical aspects, and then, if the suspicion of an eating disorder becomes more substantiated, to proceed as gently as possible in arranging care. Looking at where it comes from and what can be improved for the patient. (A2)

### Challenges and limitations in diagnosis and treatment of older adults

Experts A1, A2, A5 and A7 agreed that the diagnosis and treatment of EDs is usually complex and often goes beyond the expertise of GPs. Two participants also suspected a significantly higher number of unreported cases of older people with EDs: *Because you generally don’t have it on your radar that these things exist. There is probably a high number of unreported cases that are not even diagnosed. (A5)*

GPs also emphasize the challenge of accurately diagnosing EDs in older adults, as symptoms such as loss of appetite and weight loss often arise from various underlying health conditions, making it difficult to distinguish these disorders from other psychiatric illnesses or cancer.

Yes, it’s also harder to differentiate from malnutrition in old age, from depression, from anxiety disorder, because if I’m afraid to go shopping, then I have nothing to eat at home, plain and simple. … The appetite no longer feels so strong, let alone the feeling of thirst. So not everyone who is old and thin is anorexic. (A7)

Whether you recognise it as a doctor, because that is very suspicious in the direction of a consuming disease. It’s certainly not so easy to recognise that it’s an eating disorder, especially at an older age. (A5)

Eventually experts acknowledged that treatment strategies for older patients with ED often need to be adapted, as these individuals may have a longer history of previous therapies compared to younger patients.

So, anorexia in older age is still rare at the moment, but of course, it’s becoming more common. They’re getting older too - the young ones are growing old eventually - but there are already plenty of cases of binge eating disorder and also bulimia. But anorexia, from my perception, is still rather uncommon at this point. (A8)

## Discussion

This study explored how GPs in Austria perceive and assess patients aged 65 and older with symptoms of EDs, focusing on awareness, diagnostic approaches, and potential treatment pathways. The findings reveal substantial diagnostic challenges, the persistence of age- and gender-based stereotypes, and a marked lack of tailored clinical resources for this patient group.

### Diagnostic challenges and underrecognition of EDs in older adults

A central finding of this study was the considerable difficulty many GPs reported in recognising and diagnosing EDs in older adults. Despite recent evidence indicating that EDs are an increasing concern across the lifespan [23], several GPs described having limited or no encounters with patients over 65 presenting with ED symptoms. This perceived absence likely reflects not a genuine rarity of EDs in later life, but a longstanding pattern of underrecognition reinforced by both clinical heuristics and systemic factors in diagnostic frameworks. Consistent with earlier findings [13], GPs in this study often interpreted symptoms such as appetite loss, weight loss, and general frailty in older adults as natural consequences of ageing or attributed them to medical conditions such as cancer, gastrointestinal disease, or neurodegeneration. While it is clinically reasonable to first rule out some of these medical conditions, this approach may also reflect the tendency for mental health concerns to receive less attention at the outset of treatment (e.g., [58]), potentially delaying the recognition and management of EDs in older adults. As one participant pointed out, the complexity of multimorbidity in older age and the subtlety of ED symptom presentation can obscure the underlying disorder.

The possible co-occurrence of EDs and depression in older adults adds another layer of diagnostic complexity. In the current study, few GPs mentioned the potential interaction of ED-related symptoms and depression in later life, which aligns with previous research highlighting nuanced relationships between specific EDs and depressive symptoms.

Notably, Calvo-Rivera et al. [59] found a bidirectional association between Anorexia Nervosa and Depressive Disorder, suggesting that depression may both precede and result from Anorexia Nervosa. Shared vulnerability factors such as high self-criticism, body image distortion, and neurochemical imbalances (e.g., cortisol and oxytocin dysregulation) contribute to this interplay, with depression often persisting even after weight restoration.

Araujo et al. [60] examined the relationship between Binge Eating Disorder and depression, identifying a strong correlation but not confirming bidirectionality. Their review highlighted overlapping features such as emotional dysregulation, impulsivity, and low self-esteem, yet emphasized that most studies were cross-sectional and lacked longitudinal data to establish causality. The study findings suggest that some GPs may prioritize depression as the primary diagnosis, which may inadvertently obscure the presence of an underlying ED - particularly in cases where depressive symptoms dominate the clinical picture but may stem from or coexist with an undiagnosed ED.

GPs’ accounts further acknowledged gender-specific differences in the prevalence of EDs, particularly in older age, with the assumption that primarily older women are (or can be) affected. Epidemiological data partly support this observation, showing that although the lifetime risk for EDs generally decreases with age, Binge Eating-related disorders and Other Specified Feeding or Eating Disorders become more prevalent during perimenopause. Mangweth-Matzek et al. [12] report increased onset and symptom severity in women aged 40–60, with Binge Eating Disorder more common than restrictive types such as Anorexia Nervosa or Bulimia Nervosa. These shifts appear linked to hormonal fluctuations rather than absolute estrogen levels, potentially interacting with midlife stressors like body dissatisfaction or caregiving roles.

Such evidence suggests overlapping biological and psychosocial risk factors for Binge Eating Disorder, Other Specified Feeding or Eating Disorders, and Depressive Disorder in midlife, particularly involving hormonal and stress-related mechanisms. While Anorexia Nervosa does not show a midlife peak, it remains clinically relevant due to its association with depressive symptoms and shared neuroendocrine and cognitive-affective vulnerabilities [12]. The variability of these associations underscores the need for diagnostic specificity in research and clinical practice. Despite emerging evidence of increased symptom severity and onset of binge-type disorders during perimenopause, these patterns remain underrecognized, contributing to missed or delayed diagnoses in older women presenting with appetite or weight changes. Moreover, research on EDs beyond the reproductive years is still limited, leaving substantial gaps in understanding the trajectories, risk factors, and clinical needs of this population.

Most GPs perceived males in general, and older men in particular, as largely invulnerable to developing an ED. This perception aligns with longstanding gendered assumptions that frame EDs as predominantly female conditions, contributing to a lack of clinical awareness regarding their occurrence in men [61]. While prevalence estimates for EDs in males range between less than 1% to 3% [12, 28], this population remains significantly underdiagnosed [61]. Some GPs referred to related concepts such as muscle dysmorphia or the so-called “Adonis Complex” [62], yet these conditions were often seen as separate from typical ED presentations. Their alignment with cultural ideals of strength and muscularity may obscure pathological aspects, leading to delayed or missed diagnoses [61, 63]. Individuals with such conditions often present as fit or disciplined, while privately struggling with body image concerns and compulsive behaviours [64]. These dynamics contribute to the ongoing invisibility of disordered eating in older men and highlight the need for more gender-responsive diagnostic frameworks and clinical education.

Furthermore, psychiatric classification systems appear to have contributed to the invisibility of EDs in older adults. In DSM-4, for example, the amenorrhoea criterion for Anorexia Nervosa effectively excluded post-menopausal women [21], while many older adults presenting with disordered eating patterns were classified under the poorly studied Eating Disorder Not Otherwise Specified (EDNOS) category [26]. Although DSM-5 removed the amenorrhoea requirement and formally recognised Binge Eating Disorder, the longstanding absence of age-inclusive diagnostic criteria and the relative scarcity of research on EDNOS may have likely entrenched gaps in clinical awareness and training [21]. These structural limitations were mirrored in our interviews, where many GPs reported uncertainties regarding diagnostic tools, including limited familiarity with ED questionnaires and confusion about how to apply them effectively in older patients, alongside the absence of clear clinical guidelines tailored to this age group.

### Treatment and management of eating disorders in older adults

When it comes to treatment, GPs in this study reported a range of approaches, often influenced by perceived acceptability, time constraints, and uncertainties regarding pharmacological interventions. One GP described prescribing an antidepressant such as escitalopram (e.g., *“And maybe start with Cipralex or something like that, just to begin”* [A3]) as a pragmatic entry point, either because it was perceived as the most acceptable form of mental health treatment for older adults or as a manageable initial step when EDs were suspected but not directly addressed. Others expressed greater caution, reserving antidepressant use for what they regarded as “classic endogenous” depression without an identifiable precipitating cause. This treatment pattern may be explained by time constraints inherent to primary care consultations [65, 66], but also by a clinical preference for depression, which is viewed as more straightforward to treat than EDs [67].

The S3-Guidline for the diagnosis and treatment of EDs [68] emphasizes that pharmacotherapy alone is rarely sufficient, particularly in chronic or complex cases. For Anorexia Nervosa, the guideline recommends developing an individualized treatment plan that prioritizes quality of life, reduction of physical symptoms, and improvement of social functioning, rather than focusing solely on weight normalization. This contrasts with treatment strategies for new-onset Anorexia Nervosa, where restoring weight and eating behavior is the primary goal to prevent chronification. These distinctions are especially relevant in older adults, where multimorbidity and psychosocial factors often complicate standard treatment pathways.

Recent evidence suggests pharmacological options have varying efficacy depending on the ED subtype: Selective Serotonin Reuptake Inhibitors (SSRIs) and Tricyclic Antidepressants (TCAs) show limited benefit for Anorexia Nervosa [69], while fluoxetine may reduce depressive relapse in weight-restored patients [70]. Serotonergic agents appear to improve appetite and mood in bulimia nervosa [71]. Olanzapine in adjunct therapy with SSRIs does not lead to differences in weight gain [72], olanzapine as monotherapy can be effective in increasing weight but does not reduce depressive symptoms [73]. Lisdexamfetamine is first-line for Binge Eating Disorder [74].

Consistent with the S3 Guidelines and previous research, most GPs acknowledged that pharmacotherapy alone is often insufficient, particularly in older adults with multimorbidity. Evidence-based psychological interventions, most notably cognitive-behavioural therapy, are regarded as central components of comprehensive ED care in this population [21]. Several GPs also highlighted the importance of interdisciplinary collaboration to enhance diagnostic accuracy and support holistic management. This emphasis is well-supported by the literature: For instance, dietitians play a crucial role in nutritional rehabilitation and in addressing age-related dietary vulnerabilities [21], while occupational therapists can contribute to the restoration of daily functioning, the promotion of autonomy, and the mitigation of psychosocial consequences associated with disordered eating in later life [75]. GPs also emphasized the importance of establishing a trusting therapeutic relationship, a factor consistently highlighted as critical in the literature for effective ED management (e.g., [76, 77]).

### Gendered ageism and the invisibility of EDs in older adults

The findings of this study underscore the role of gendered ageism - the intersection of age- and gender-based biases [78] - in shaping how EDs are assessed, recognised, and treated in older adults [12, 79, 80]. Many GPs implicitly or explicitly associated EDs primarily with younger, typically female patients, reflecting broader societal discourses that frame EDs as youth-centric and largely confined to adolescence or early adulthood [1–3]. In contrast, older adults, especially women, were often characterised as experiencing self-neglect, somatic deterioration, or reduced appetite, rather than presenting with a serious psychiatric disorder. One GP, for instance, drew a clear distinction between “real eating disorders” in younger patients and what was described as “neglect” in older individuals.

While EDs in younger adults are widely recognised as serious mental health conditions, several GPs in this study tended to attribute weight loss or restrictive eating in older adults to physiological changes associated with ageing - such as general frailty, reduced appetite, or a natural decline in muscle and bone mass - rather than to psychiatric illness. These clinical impressions align with the broader research discourse on the so-called “Anorexia of Ageing” [81], a concept that frames late-life appetite and weight loss primarily as biological phenomena linked to sarcopenia, cachexia, or somatic deterioration. Such interpretations, while medically plausible, risk overlooking the psychological and behavioural dimensions of disordered eating in later life. They may also reflect broader ageist assumptions that psychological distress in older adults is a normal by-product of ageing rather than a sign of an underlying, potentially reversible condition [39]. As a result, the threshold for psychiatric referral may be inadvertently raised, and the diagnostic visibility of EDs in older adults reduced. Notably, these biases appear especially pronounced in the case of older women, whose symptoms are often dismissed or misattributed due to assumptions that they are no longer concerned with body image or appearance [78].

Moreover, other terminology in the literature, such as perimenopausal EDs [10], tends to emphasize physiological or hormonal factors, particularly changes in estrogen, while largely overlooking the psychological dimensions of disordered eating. Such interpretations reflect societal expectations linking femininity to thinness [82] while rendering older women emotionally less complex or asexual [83], further reducing diagnostic vigilance and the likelihood of psychiatric referral.

While older women are typically the focus of discussions on gendered ageism (e.g., [80, 84, 85]), this study revealed that older men with EDs are almost entirely absent from clinical awareness. Most participants reported little to no experience with male patients diagnosed with EDs, likely due to the perception of EDs as largely “female disorders”. As a result, symptoms in older men may be overlooked or misattributed to other causes such as general health decline or personality traits. Male body image concerns, when acknowledged at all, are often reframed through the earlier mentioned less pathologized constructs like muscle dysmorphia or the “Adonis Complex”. These conditions, though characterized by obsessive muscularity, body dissatisfaction, compulsive exercise, and in some cases the use of performance-enhancing substances [62, 64, 79], are frequently normalized within masculine cultural ideals and fitness discourses. Their ego-syntonic nature - meaning the behaviours are consistent with the individual’s self-image - and the appearance of health or discipline often conceal underlying pathology, reducing both self-reporting and clinical detection [61, 63].

This dual marginalization - due to both gender and age - reflects a deeper form of gendered ageism that renders older men emotionally simplistic, less vulnerable to mental health struggles, and therefore less deserving of psychiatric attention (e.g., [86, 87]). The lack of male-specific screening tools, combined with the stigma men may feel in acknowledging body dissatisfaction or emotional distress, further compounds the diagnostic gap. These structural and cultural barriers highlight the urgent need to challenge prevailing gendered narratives and incorporate older men into age- and gender-sensitive frameworks for ED research, assessment, and care.

Participants in this study exclusively referred to binary gender concepts, a pattern that mirrors much of the existing literature on gendered ageism and EDs, where non-binary and gender-diverse identities are frequently overlooked [78]. Although a growing body of research has begun to explore ED experiences among transgender and non-binary individuals [88, 89], future studies should more explicitly incorporate non-binary perspectives to advance inclusive, gender-sensitive approaches to ageing and mental health.

Taken together, the underrecognition and undertreatment of EDs in older adults cannot be attributed solely to individual clinical oversight. Rather, it reflects the interplay of diagnostic frameworks historically biased toward youth, a research base that has largely neglected later-life presentations, entrenched cultural narratives about ageing, gender and mental health, and structural conditions in primary care that prioritize rapid, pharmacologically oriented responses to depression over holistic assessments of nutritional and behavioural concerns. These systemic factors produce overlapping forms of invisibility for women and men alike, underscoring the urgent need for age- and gender-sensitive frameworks in research, screening, and clinical treatment of EDs in later life. Integrating interdisciplinary approaches and fostering strong clinician–patient relationships may help mitigate these gaps, ultimately improving recognition, care, and outcomes for this underserved population.

### Limitations

Several factors may limit the interpretability and generalizability of this study’s findings. The analysis is based on a small number of experts (*n* = 9), and due to challenges in recruiting additional GPs, thematic saturation may not have been fully achieved. While a total of 1,035 GPs were contacted, only nine ultimately participated in the interviews. Several factors may have contributed to the low response rate: the study was conducted as part of a master’s thesis, which may have influenced its perceived relevance or priority; Austria’s hierarchical healthcare system could have impacted the motivation to participate in a study led by a dietitian; and time constraints, competing clinical responsibilities, and limited availability among GPs likely further reduced participation.

Potential recruitment bias must also be considered. Since participants were informed about the study’s focus, those with prior experience or interest in EDs may have been more inclined to participate, possibly leading to an overrepresentation of certain perspectives. Additionally, one participant disclosed personal experience with an ED, which may have enhanced sensitivity to subtle symptoms but also introduced a degree of subjective bias. The limited geographic scope – restricted to two Austrian federal states – further constrained participant diversity. Finally, social desirability bias [90] may have influenced the responses: although GPs reported treating all patients equally, unconscious biases may shape clinical decision-making in ways not fully captured through self-report interviews.

### Implications and future directions

Our findings have several implications for clinical practice, research, and public health policy. First, there is a clear need for age-adapted diagnostic tools for EDs, as current instruments have not been validated for use with older adults. Future validation studies should aim to refine screening methods to detect EDs across the lifespan, accounting for both somatic and psychological dimensions. Second, treatment pathways tailored to older adults are lacking. Most existing treatment protocols are designed for adolescents or young adults, often focusing on body image-related concerns that may be less central for older individuals. More nuanced, flexible, and age-sensitive approaches are required, ideally integrating somatic care, mental health support, and social interventions. Third, raising public and professional awareness is essential. EDs in older adults continue to be underrecognised not only in primary care but also in broader societal discourse. Public health campaigns should communicate that EDs can occur at any age and in any gender, countering persistent myths and stigmas.

From a research perspective, a life course approach to EDs would enable a more integrated understanding of how disordered eating may manifest differently across the lifespan. This could include longitudinal research, as well as studies investigating how earlier-life experiences interact with late-life stressors. Although the primary aim of this study was to explore diagnostic and treatment approaches for EDs in later life, it also engaged with the possible influence of ageism and gendered ageism. While this focus remained exploratory and inductive, future quantitative studies could test the hypothesis that older individuals with ED symptoms are assessed or treated differently from younger patients, for instance, by employing contrastive vignette techniques. In this context, it is also important to consider the potential role of social desirability bias [90]. GPs may not consciously recognise that they treat older patients differently, but in an interview setting, they are likely to emphasize equal treatment, as acknowledging age-based distinctions may be perceived as discriminatory. Future experimental designs could help to control for such biases and provide a more objective assessment of age-related disparities in diagnosis and care. Finally, and most importantly, the current study did not include perspectives from individuals over 65 years old who are directly affected by EDs, resulting in the omission of essential firsthand experiences. This highlights the need for future research to explore the lived experiences of older adults with EDs in order to inform more responsive clinical practices and care strategies.

## Supplementary Information

Below is the link to the electronic supplementary material.

Supplementary Material 1

Supplementary Material 2

## Data Availability

Data extracted from the articles included in the current study are available from the corresponding author upon reasonable request.
