# Supplementary material for: Overlooked and undertreated: gendered ageism in primary care management of eating disorders
Source: Int J Equity Health. 2025 Dec 30;24:358. doi: 10.1186/s12939-025-02702-0 (PMC12754874; doi:10.1186/s12939-025-02702-0)
Supplement: Supplementary file 1 — Supplementary Material 1 [file 12939_2025_2702_MOESM1_ESM.pdf]

Interview Guide

Warm-up

- Welcome, acknowledgements, introduction of researcher
- Clarification of framework conditions, procedure, questions, wishes, concerns
- Signing of the consent form

START OF AUDIO RECORDING

Interview

Personal details

- Age
- Gender
- Professional background or brief description of current professional activity (how long, individual or group practice, etc.)

Vignette is given to the interviewee to read through

Now that you have read through this vignette, what are your initial thoughts?

a. While reading, did you think of a specific patient from your practice, or are you currently treating someone with similar symptoms?
b. If so, can you describe this person in general terms and what the treatment strategy was?
c. If not, based on your experience, what would a treatment strategy for such a patient look like?

What is your experience with older patients with eating disorders?

a. How often do you encounter elderly patients with eating disorders?
b. How would you describe the relationship between you and this patient group?
c. Do you have experience with young people with eating disorders?
d. If so, how do the symptoms differ between older and younger patients?
e. If not, what differences do you think could occur in the symptoms between older and younger patients?

Do you have any experience with gender-specific differences in the disease itself, but also in its diagnosis?

a. If so, how do these differences manifest themselves?
b. If not, how do you think these differences might manifest themselves?

Could you describe the course of treatment for a person over 65 with an eating disorder?

a. Is the course different from that of a younger person?

b. What limitations might you encounter, for example? (e.g. in relation to diagnosis, treatment or adherence to therapy)
c. Are the resources available for this sufficient, or would you like to see something else? (e.g. assessment, screening, guidelines, further training, dietitian)

Conclusion

- Is there anything else you would like to say on this topic?

- Clarify any open questions, statements, possible feedback

- Thank you, farewell

END OF RECORDING

Vignette

Name: Elisabeth Müller

Age: 67 years old

Gender: Female

Height: 165 cm

Professional background: Retired, former teacher (sports and mathematics)

Marital status: Widowed, two adult children

Living situation: Lives alone in a city flat

Medical history:

Generalised anxiety disorder

Depression

Hypothyroidism

Osteoporosis

Gastroesophageal reflux disease with oesophagitis

Hypotension and bradycardia

Mrs Müller complains of increasing weakness and dizziness when using the stairs to her flat (3rd floor). She has always been very active and athletic and would like to integrate more regular exercise into her daily routine again. The patient appears noticeably thin and, when asked about this, mentions that she lost weight after her husband's death six months ago. When asked further, she states that she has lost weight from approximately 55 kg to 41 kg. Ms Müller says that she eats healthily and watches her calorie intake, which is very important to her. She also mentions increased hair loss, low blood pressure, difficulty concentrating and past bone fractures due to osteoporosis.
